# Structural changes and mechanisms of reversible electrochemical lithium-ion cycling in (104) oriented LiNi_1/3_Mn_1/3_Co_1/3_O_2_ thin film cathodes prepared by pulsed laser deposition†

**DOI:** 10.1039/d4ra08924c

**Published:** 2025-04-07

**Authors:** Blaž Jaklič, Jan Žuntar, Elena Tchernychova, Gregor Kapun, Martin Šala, Robert Dominko, Matjaž Spreitzer

**Affiliations:** a Advanced Materials Department, Jožef Stefan Institute Jamova cesta 39 1000 Ljubljana Slovenia blaz.jaklic@ijs.si; b Jožef Stefan International Postgraduate School Jamova cesta 39 1000 Ljubljana Slovenia; c National Institute of Chemistry Hajdrihova ulica 19 1000 Ljubljana Slovenia; d Faculty of Chemistry and Chemical Technology, University of Ljubljana Večna cesta 13 1000 Ljubljana Slovenia; e Alistore-European Research Institute, CNRS FR 3104, Hub de l'Energie Rue Baudelocque 80039, Amiens France

## Abstract

This study examines the structural and electrochemical behavior of epitaxial (104) oriented LiNi_1/3_Mn_1/3_Co_1/3_O_2_ (NMC 111) thin film cathodes prepared by pulsed laser deposition, aiming to elucidate the underlying mechanisms of reversible lithium-ion cycling. The effect of growth parameters on film morphology and crystal structure is thoroughly studied. The surface analysis confirms the oxidation states of transition metal ions to be Ni^2+^, Mn^4+^ and Co^3+^. Microstructural analysis reveals twinned domains in the NMC 111 layered structure, which conforms with its 4-domain crystallographic orientation. After NMC 111 thin film is charged to 4.2 V, a change in the local electronic structure of nickel and oxygen ions is observed by electron energy loss spectroscopy as a consequence of nickel oxidation. By utilizing *ex situ* reciprocal space mapping after charging to 4.2 V, a negative unit cell volume change was observed, compensated by an increased mosaic spread of NMC 111 lattice planes. This structural adjustment is reversibly maintained upon discharging to 3.0 V. Based on defined epitaxial structures, the reversible mechanism of lithiation and delithiation in NMC 111 thin films is determined on a structural level, providing detailed insight into its functionality. To address structural instability in the charged state, the electrochemical performance was enhanced by cooling the NMC 111 thin films under high oxygen pressure.

## Abstract 2

(104)-Oriented epitaxial NMC thin film cathodes exhibited negative volume change, increased mosaicity and nickel oxidation when charged to 4.2 V.

## Graphical abstract

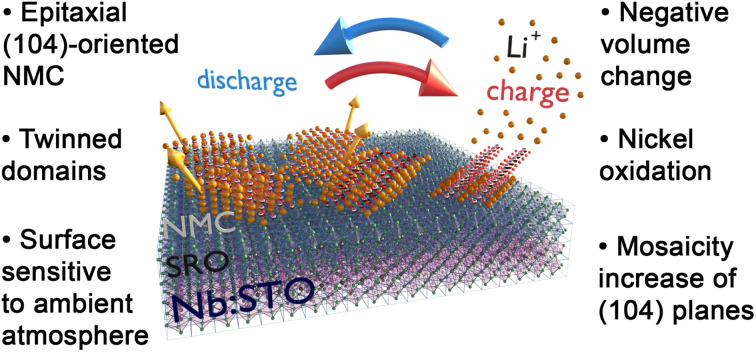

## 1. Introduction

One of the promising cathode materials for lithium-ion batteries is LiNi_1−*x*−*y*_Mn_*x*_Co_*y*_O_2_ (NMC) because of its high specific capacity, thermal stability and safety, as each transition metal in the structure has unique properties. Nickel ions contribute to high specific capacity, cobalt ions give high rate capabilities and manganese ions stabilize the structure because they are strictly in a 4+ oxidation state.^1^ However, NMC cathodes suffer from performance degradation that is linked to the instability of the material in the charged state. Structural instability of NMC at higher voltages can cause spontaneous reduction of Ni^4+^ ions to more stable Ni^2+^. The presence of Ni^2+^ in a charged cathode causes the migration of divalent nickel to lithium interstitials. The reason for cation disordering is a similarity in ionic radius of Ni^2+^ (0.69 Å) and Li^+^ (0.76 Å), so the cation mixing degree increases with nickel content, state of charge, and temperature.^2–5^ Consequently, NMC is exposed to structural changes and phase transitions from initial layered over spinel to NiO-like rock salt structure. Furthermore, structural instability is strongly related to oxygen non-stoichiometry, since oxygen vacancies initiate additional oxygen loss from the surface during the charge/discharge process and give rise to cation disorder on the surface of charged NMC cathode, so controlling the oxygen stoichiometry in NMC is crucial.^6^

Thin film electrodes, prepared *via* pulsed laser deposition (PLD) can act as a model system for studying the intrinsic properties of battery materials and their interfaces, but it is necessary to carefully control synthesis parameters to prepare high-quality epitaxial thin films.^7–9^ Furthermore, oriented thin films can provide useful information on the anisotropic material properties since no binder and conductive additives are present in thin film electrodes. Several groups reported the growth of NMC thin films by PLD,^10–18^ of which using single crystal oxide substrates proved to be most successful in the preparation of epitaxial NMC thin films.^10,15–17^ Hirayama *et al.* studied the influence of crystal orientation of SrRuO_3_ (SRO)/Nb:SrTiO_3_ (Nb:STO) substrate on the growth of NMC 111 film. Films with preferred out-of-plane crystal orientations of (104),(11̄8), and (003) were deposited on SRO/Nb:STO substrates with out-of-plane crystal orientations of (100), (110), and (111), respectively. Electrochemical measurements of this study revealed the anisotropic properties of NMC 111 cathodes as (104) surface exhibited reversible behavior at deep cycling to 4.5 V, but the (11̄8) and (003) surface planes showed fading of average discharge voltage and specific capacity.^15^ Moreover, a detailed analysis of the growth mechanism and the structural properties of epitaxial layered oxide thin films revealed a 4-domain crystallographic orientation of the films on (100) oriented STO substrates for both NMC^15,16^ and LiCoO_2_ (LCO).^19–21^ However, the microstructural evolution of epitaxial layered thin film cathodes during cycling was reported only for LCO (003), where TEM observations showed the relaxation of translational domain boundaries during cycling, while Electron Energy loss spectroscopy (EELS) suggested the reduction of cobalt ions on the surface, which impeded lithium ion insertion during discharging.^22^ With this in mind, the structural evolution of twinned domains and charge compensation mechanisms in NMC with (104) preferred orientation warrants further investigation, given the superior electrochemical properties demonstrated by such layered cathode thin films.^23^

In this study, we report the reversible mechanism of delithiation and lithiation on the structural level in (104) out-of-plane oriented NMC 111 thin films, as (104) surfaces of lithium layered oxides proved to be thermodynamically favorable and most stable electrochemically.^24^ The structural properties of NMC 111 thin films were studied in detail by HRXRD, proving the 4-domain in-plane crystallographic orientation and relaxation of the unit cell on SRO. Microstructural analysis revealed twinned domains and the presence of a rock-salt phase on the surface of the thin film while surface analysis revealed the sensitivity of NMC 111 thin films, exposed to ambient conditions. *Ex situ* HRXRD analysis showed how the structure of NMC 111 thin film evolves when lithium is removed from the structure, resulting in reversible negative volume change that is compensated by increased mosaicity in the thin film cathode at the charged state. The change in the local electronic structure of nickel and oxygen ions was observed by EELS, explaining the charge compensation mechanism by nickel oxidation. Thin films, prepared under different oxygen environments demonstrated that oxygen background pressure during growth and cooling plays an important role in the morphological and electrochemical properties of NMC 111. The overall electrochemical response is revealed by performing cyclic voltammetry and galvanostatic cycling at different current densities.

## 2. Experimental methods

### 2.1. Target and sample preparation

LiNi_0.33_Mn_0.33_Co_0.33_O_2_ target with 30% lithium excess was prepared in-house by conventional solid-state synthesis route. To obtain stoichiometric NMC 111 compound, Li_2_CO_3_ (Puratronic, 99.998%, 4% excess), NiO (Sigma-Aldrich, 99.99%), MnO_2_ (Thermo Fischer, 99.9%), and Co_3_O_4_ (Puratronic, 99.9985%) powders were mixed and dispersed in acetone. After the ball-milling (1 h, 250 rpm) and drying (30 min, 80 °C), powders were calcined two times at 800 °C for 12 h. Powders were uniaxially pressed to pellets (200 MPa) before firings. The as-prepared stoichiometric NMC 111 powder was mixed with a 15% molar excess of Li_2_CO_3_, to compensate for lithium loss during thin film synthesis. Finally, NMC powder with Li_2_CO_3_ excess was pressed with an isostatic press (700 MPa) and sintered at 850 °C for 12 h to prepare the PLD target. SrRuO_3_ (99.95%) target was purchased from Beijing Goodwill Metal Technology.

NMC 111 thin films were synthesized using a PLD system from Twente Solid State Technology equipped with a 248 nm ultraviolet KrF excimer laser (Coherent COMPex 205) with a 20 ns pulse. Films were grown on 0,5%wt Nb-doped SrTiO_3_ (001) single crystals (CrysTec). Before NMC deposition, SrRuO_3_ film was grown epitaxially on Nb:STO (001). The spot size of the ablated area was fixed at 2.31 mm^2^ and the target-to-substrate distance was fixed at 55 mm for all depositions. For SrRuO_3_ deposition, the temperature of the resistive heater was set to 585 °C, the oxygen pressure in the chamber was 0.13 mbar and the laser fluence was fixed to 2.5 J cm^−2^. The ablation frequency and the number of laser pulses were 4 Hz and 3500 pulses, respectively. For NMC 111 thin film deposition the temperature of the resistive heater was set to 600 °C, the laser frequency was 5 Hz and the laser fluence was fixed to 1.5 J cm^−2^. The oxygen pressure in the PLD chamber during growth varied in ranges from 10^−5^ mbar to 0.25 mbar. Cooling oxygen pressure was the same as deposition pressure or 500 mbar for annealed samples. The heating and cooling rate was set to 10 °C min^−1^. Reflection high-energy electron diffraction (RHEED) was used to monitor surface structure changes and thin film growth during the deposition.

### 2.2. Structural and surface characterization

The *θ* − 2*θ* patterns, azimuthal *φ* patterns, reciprocal space maps (RSM), and X-ray reflectometry (XRR) measurements were collected with an X-ray diffractometer (Empyrean, Malvern PANalytical) with CuKα_1_ radiation (*λ* = 1.5406 Å). A double-bounce Ge (220) hybrid monochromator was used on the incident-beam side. A PIXcel3D detector operating in 1D mode captured and analyzed the diffracted beam. In azimuthal *φ* scans, *χ* was set to 45° and 55.15° to align STO (110) and NMC (003) planes with the X-ray beam, respectively. For RSMs, sample was aligned to Nb:STO 
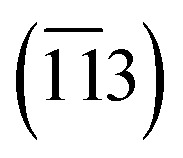
 planes before the measurement. The values were converted from angular units to reciprocal space coordinates *Q* (*Q*_*x*_ for the in-plane component, *Q*_*y*_ for the out-of-plane component) using equations *Q*_*x*_ = *R*(cos *ω* − cos(2*θ* − *ω*)) and *Q*_*y*_ = *R*(sin *ω* + sin(2*θ* − *ω*)), where *R* = 0.5. The results are presented in the form of contour plots of intensity *versus Q* (*Q*_*x*_ and *Q*_*y*_) in the reciprocal lattice unit (r.l.u.). A parallel plate collimator was used at the diffracted side for X-ray reflectometry measurements. The thickness of the films was estimated from XRR measurements by the Fourier method.

TEM lamella sample preparation was performed by using a FIB Helios Nanolab 650 (Thermo Fisher Scientific, The Netherlands). Sample and FIB lift-out grid was mounted on a vacuum transfer shuttle inside the Ar-filled glovebox and transferred directly to the FIB instrument without being exposed to air atmosphere. The sample surface was initially protected by 3 nm of carbon and 300 nm Pt layer by using electron beam-induced deposition (EBID, 2 kV @ 0.4 nA). Subsequently, an additional Pt layer was deposited by using Ga^+^ ion beam-induced deposition (IBID, 30 kV @ 0.23 nA) to achieve a protective layer with a final thickness of 1.5 μm. A rough lamella chunk with dimensions of 12 × 6 μm was milled perpendicular to [110] direction of Nb:STO substrate, thinned to 2 μm and transferred to the FIB grid using a micromanipulator. Due to sample sensitivity, lamella was first thinned to 250 nm thickness using FIB at 30 kV by sequential reducing ion beam currents from 780 pA to 80 pA. Subsequently, lamella was carefully thinned to 100 nm using FIB at 16 kV with 20 pA beam current. Afterward, lamella was sequentially polished on both sides using FIB at 5 kV @ 44 pA and 2 kV @ 25 pA until electron transparency (∼50 nm). As prepared lamella sample was transferred with a vacuum transfer system directly from the FIB chamber to the glovebox where it was mounted to the TEM vacuum transfer holder. The cross-sectional samples of interfaces were examined by a JEM-ARM200CF probe Cs-corrected scanning transmission electron microscope (STEM) equipped with a cold field emission electron source operated at 80 kV. EELS analysis was performed using a QuantumGIF imaging filter (GATAN, Plesanton, U.S.A.) attached to the JEM-ARM200CF probe Cs-corrected microscope. The 2D spectrum images with 0.75 eV energy resolution, 0.25 eV energy dispersion, 130 × 1 binning and 0.1s pixel time were recorded from the films' cross-sectional FIB lamellae. The analyzed EEL spectra were then extracted at the bulk of the film by summing up the pixels in lines parallel to the substrate, excluding the surface and the interface to compare pristine and charged EEL spectra of NMC 111 thin film. The changes in oxidation states of TMs were then determined by using a modified integral Mn, Ni and Co L3,2 white-line intensity ratios,^25^ calculated for each line sum spectrum. Simulated selected area diffraction patterns were obtained with the use of CrystalMaker Software.

X-ray photoelectron spectroscopy (XPS) was performed with the Versaprobe 3 AD (Phi, Chanhassen, US) using a monochromatic Al-Kα_1_ X-ray source. For each measurement, spectra were acquired on a 200 μm spot size with the charge neutralizer turned on, as the films/powders were put on a non-conductive double tape to prevent possible differential charging of lithium-containing material. High-resolution spectra were measured at 55 eV pass energy and step of 0.05 eV. Charge neutralization was used, so the energy scale of photoelectron spectra was corrected by shifting the C 1s peak of carbon to the binding energy of 284.8 eV. To clean the surface layer from carbon contamination, sputtering of the sample with an argon gas cluster ion beam (GCIB) was used. The film was cleaned with GCIB operating at 10 kV @ 30 nA over 2 mm × 2 mm area. After cleaning of the surface with GCIB, sputtering with argon ions was utilized operating at 3 kV over 2 mm × 2 mm area to remove lithium residuals. Since the shift of the spectra to C 1s after sputtering was not possible, spectra were shifted to O 1s peak of lattice oxygen in NMC 111 to the binding energy of 529.3 eV. XPS spectra were analyzed with PHI Multipak software. For the fits, the error in the binding energy scale for all peaks was limited to ±0.2 eV. Shirley background correction was used for all the spectra.

Atomic force microscopy (AFM) was performed with Veeco Dimension 3100 SPM to study the surface morphology of the films. AFM images were analyzed and edited with WSxM 5.0 software.^26^

Inductively coupled plasma-optical emission spectroscopy (ICP-OES) was used for the elemental analysis of bulk ceramic powders, while inductively coupled plasma-mass spectroscopy (ICP-MS) was used for the elemental analysis of thin films. All reagents were of analytical grade. For sample dilution and preparation of standards, ultrapure water (18.2 MΩ cm^−1^, Milli-Q, Millipore) and ultrapure acids (HNO_3_ and HCl, Merck-Suprapure) were used. Standards were prepared in-house by dilution of certified, traceable, inductively coupled plasma (ICP)-grade single-element standards (Merck CertiPUR). Before the ICP-OES analysis of bulk ceramics, each sample was weighed (approximately 10 mg) and digested by dissolving it in concentrated HCl (5 ml). Samples were then diluted with 2% vol. HNO_3_ until the concentration was within the desired concentration range. Before the ICP-MS analysis of thin films, the sample was digested in 2 ml of aqua regia at 100 °C for 30 minutes. The digested sample was cooled to room temperature and then diluted with 2% vol. HNO_3_ until the concentration was within the desired concentration range.

### 2.3. Electrochemical characterization

CR2032 type coin cells were assembled in the Ar-atmosphere glovebox with the inner stack consisting of 6 mm precycled Li_4_Ti_5_O_12_ (LTO) electrodes, Celgard™ 2320 separator (18 mm in diameter) and 10 × 10 × 0.5 mm thin-film NMC electrodes, with 15 μL of LP40 (1 M LiPF_6_ in EC/DEC = 1 : 1 vol., Sigma-Aldrich) being added as the electrolyte. The preparation of precycled LTO counter electrodes is described in the ESI file.† To improve charge transfer between the substrate and the stainless steel disk of the coin cell, gold was sputtered on the backside of the substrate before the assembly of the thin film battery. All electrochemical characterization was performed with a BioLogic VMP3 multichannel potentiostat. The coin cells were galvanostatically cycled in the range of 0.92–2.6 V *vs.* LTO voltage plateau (roughly corresponding to 2.5–4.2 V *vs.* Li/Li^+^) with a current density of 0.5 μA cm^−2^ (C/8 rate).

To study the structural changes taking place in NMC 111 thin film electrodes with respect to the degree of lithiation of the material, NMC|Li liquid electrolyte pouch cells were assembled inside an Ar-atmosphere glove box and electrochemically analyzed. For the pouch cell assembly step, 12 mm disks were punched out of 110 μm thick lithium foil (FMC Corporation) and gently brushed with a plastic cylinder to obtain a fresh metal surface. Additionally, 18 mm disks were punched out of 260 μm thick glass fiber paper (Whatman, GF/A glass microfiber) and used as a separator. LP40 was used as the electrolyte, with 7 μL distributed on the Li foil and 75 μL on the GF/A separator, amounting to a total electrolyte volume of 82 μL. The pouch cell was made of polypropylene/polyethylene/polypropylene laminated Al foil, with thin metal foil strips serving as contacts (Al foil for the NMC electrode, and Cu foil for lithium). After each set of electrochemical measurements, the pouch cell was disassembled inside a glove box, where the delithiated NMC electrode was washed with diethyl carbonate (DEC, Sigma Aldrich) and stored for XRD analysis or FIB lamella preparation. The subsequent reassembly of the pouch cell with the analyzed NMC electrode was performed identically as described above.

#### 2.3.1 Delithiation

To form a stable interface, the pouch cell was first slowly charged to 4.2 V *vs.* Li/Li^+^ with a current density of 0.12 μA cm^−2^, corresponding to a real charging time of 120 h. After the first charging step, the cell then underwent one galvanostatic discharge–charge cycle in the range of 3.0–4.2 V *vs.* Li/Li^+^ at a higher current density of 0.8 μA cm^−2^ (corresponding to an effective C-rate of C/5), which was followed by approximately 10 h of relaxation at OCV.

#### 2.3.2 Lithiation

The pouch cell was first discharged to 3.0 V *vs.* Li/Li^+^ with a current density of 0.2 μA cm^−2^, corresponding to a real discharge time of approximately 8 h. In a similar fashion to the delithiation procedure, the cell then underwent one galvanostatic charge–discharge cycle in the range of 4.2–3.0 V *vs.* Li/Li^+^ at 0.8 μA cm^−2^ (corresponding to an effective C-rate of roughly C/5), which was finally followed by approximately 10 h of relaxation at OCV.

#### 2.3.3 Rate testing

To investigate the electrochemical performance of NMC 111 thin film electrodes under different charging/discharging currents, a rate testing program was performed on NMC|Li half-cells in the pouch cell configuration. The cells were first slowly charged to 4.2 V *vs.* Li/Li^+^ with a current density of 0.2 μA cm^−2^ and then underwent two discharge–charge cycles in the range of 3.0–4.2 V *vs.* Li/Li^+^ at 0.4 μA cm^−2^ (C/10), ensuring the formation of a stable interface. Afterwards, three discharge–charge cycles were performed at current densities of 2 μA cm^−2^, 4 μA cm^−2^, 8 μA cm^−2^, 16 μA cm^−2^ and 24 μA cm^−2^, corresponding to C-rates of C/2, 1C, 2C, 4C and 6C, respectively.

#### 2.3.4 Cyclic voltammetry

Cyclic voltammetry measurements were performed in the range of 3.0–4.2 V *vs.* Li/Li^+^ with a sweep rate of 0.1 mV s^−1^.

## 3. Results and discussion

### 3.1. Target structure and stoichiometry

**Fig. 1:**
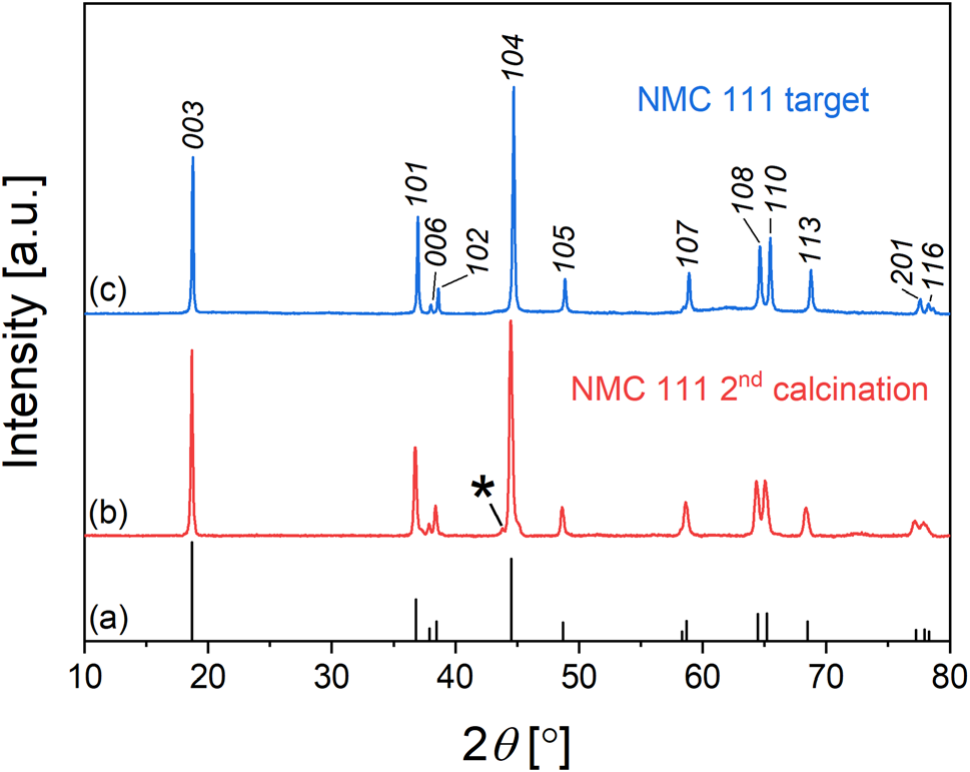
XRD pattern of (a) NMC 111 reference, (b) NMC 111 powder after 2nd calcination and (c) NMC 111 PLD target. * NMC spinel impurity peak.

The X-ray diffraction (XRD) patterns of the calcined NMC 111 powder and NMC 111 target with lithium excess are shown in Fig. 1. All diffraction peaks can be indexed to polycrystalline reflections of layered *R*3̄*m* space group indicating a single crystalline phase in both powders, with minor NMC spinel impurity in the calcined powder. The stoichiometry and molar ratios between lithium, nickel, manganese and cobalt determined by ICP-OES were calculated to be Li_0.98±0.01_Ni_0.33±0.01_Mn_0.35±0.01_Co_0.32±0.01_O_2−*γ*_ for calcined powder and Li_1.21±0.01_Ni_0.33±0.01_Mn_0.35±0.01_Co_0.32±0.01_O_2−*γ*_ for NMC 111 target. Calcined NMC 111 powder was close to stoichiometric, while NMC 111 target with 30 mol% lithium excess added during synthesis exhibited 21 mol% lithium excess after sintering, indicating some lithium evaporated at elevated temperatures during the sintering of the target.

### 3.2. Structural analysis of LiNi_1/3_Mn_1/3_Co_1/3_O_2_ thin films

**Fig. 2:**
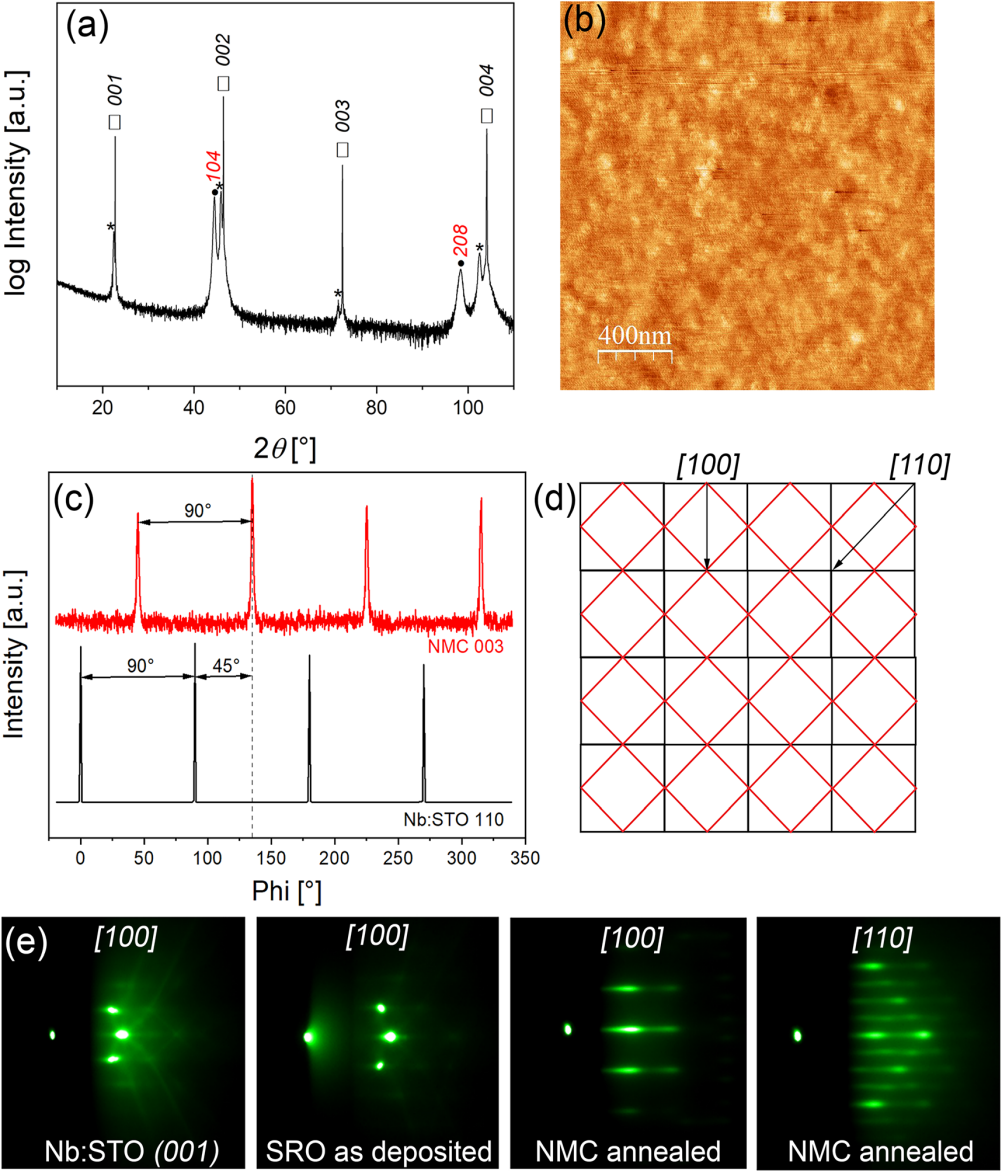
(a) Out-of-plane XRD pattern of NMC 111 thin film on SrRuO_3_/Nb:SrTiO_3_ (001) substrate. * SrRuO_3_ peaks, □ Nb:SrTiO_3_ peaks, • NMC 111 peaks; (b) AFM image of NMC 111 thin film, deposited at 0.01 mbar and annealed at 500 mbar; (c) Azimuthal *φ* patterns of NMC 111 (003) and Nb:SrTiO_3_ (110) reflections; (d) graphical presentation of in-plane symmetry relation between NMC 111 thin film (red) and the substrate (black); (e) *in situ* monitoring of surface structure with RHEED.

XRD pattern of NMC 111 thin film on the SRO/Nb:STO (001) is shown in Fig. 2a. The diffraction peaks can be attributed to the substrate, SRO and two NMC 111 peaks at about 44.5° and 98.4°, corresponding to (104) and (208) out-of-plane orientation of the film, respectively, indicating epitaxial growth of NMC 111 on SRO/Nb:STO (001) substrate. A comparison of XRD patterns for NMC 111 thin films, deposited at different oxygen pressures is shown in Fig. S1.† Deposition at different oxygen pressures influences the structure and that is reflected as a shift of (104) peak from the original position. The bulk value for *d*-spacing of (104) reflection was obtained at 0.01 mbar and annealed at 500 mbar oxygen pressure. Annealing of NMC 111 thin films in high oxygen pressure can initiate additional oxygen incorporation in the films, thus stabilizing the crystal structure of NMC 111. Since samples annealed in an oxygen-rich environment showed improved electrochemical performance of thin films, all structural properties were studied on those samples, respectively. Fig. 2c depicts an azimuthal *φ* scan on the (110) plane of the substrate and (003) plane of the NMC 111 film. This result proposed the pseudocubic growth of NMC 111 film on SRO/Nb:STO (001) substrate with 4-fold symmetry and 45° in-plane angle tilt to the substrate (Fig. 2d), which agrees with the literature reports on the growth of NMC on STO.^15,16^ RHEED patterns of Nb:STO substrate, as-deposited SRO and NMC 111 after annealing recorded in [100] azimuth direction indicate the epitaxial flat surface of as-deposited SRO and the slightly stepped surface of annealed NMC 111 (Fig. 2e). Additional RHEED pattern of NMC 111 thin film after annealing, recorded in [110] azimuth direction shows slightly modulated streaks, confirming multilevel stepped terraces on the surface.^27^ The morphology of pristine NMC 111 thin film, determined by atomic force microscope (Fig. 2b), showed a smooth flat surface with the RMS roughness value of 0.36 nm. A comparison of NMC 111 thin films, prepared in different oxygen environments, taken from the 2 μm × 2 μm area, is shown in Fig. S2.† It is evident that the films get porous at higher deposition pressures (0.25 mbar), while a morphology difference is observed when the films are annealed in high oxygen pressure after deposition, resulting in a smoother surface of dense films, so the NMC 111 thin films should be grown at lower pressures and cooled in high pressures to prepare compact and dense films with a flat surface.

The thickness of the SRO bottom electrode and NMC 111 thin films was estimated by XRR using the Fourier method to be 35 nm ± 3 nm for SRO and 39 nm ± 2 nm, 45 nm ± 2 nm, 72 nm ± 3 nm and 79 nm ± 3 nm for NMC 111 deposited at 0.25 mbar, 0.1 mbar, 0.01 mbar and 10^−5^ mbar oxygen pressures, respectively (Fig. S3†). The growth rate of the film decreases with increasing oxygen pressure, since the kinetic energy of the incoming species in the plasma plume is slowed down by the background gas, so the film grows slower at high pressures. The stoichiometry of NMC 111 thin film, deposited at 0.01 mbar and subsequently annealed was estimated by ICP-MS to be Li_1.00±0.05_Ni_0.32±0.01_Mn_0.28±0.01_Co_0.34±0.01_O_2−*γ*_. Comparison between the stoichiometry of the PLD target and thin film confirmed some lithium loss during the deposition process, but since lithium excess was added to the target, the composition of the thin film was close to the stoichiometric.

A symmetrical RSM collected around the Nb:STO (002) reflection of a substrate is shown in Fig. S4a.† The broad reflection of the (104) peak indicates large mosaicity in the NMC 111 thin film due to 4-domain crystallographic orientations. To study the in-plane orientation relationship between SRO/Nb:STO (001) substrate and NMC 111 film, RSMs were collected around Nb:STO 
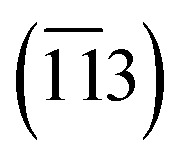
 reflection with the presence of NMC 111 (1 0 10) and (202) reflections (Fig. S4b†). The in-plane reciprocal direction *Q*_*x*_ of the substrate and the film are far apart, indicating that the film is relaxed. Since SRO 
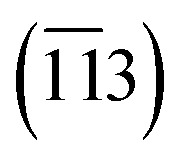
 is a forbidden reflection, additional RSM was performed around Nb:STO (01̄3) to confirm that SRO is coherently strained to the substrate (Fig. S4c†). The relaxation of NMC 111 is a consequence of a large lattice mismatch (>4%) between the film and strained SRO in the in-plane direction. The lattice parameters and volume of the hexagonal NMC 111 unit cell were calculated from RSMs, using equations described in the ESI file.† Calculated parameters from RSMs were 2.88 Å for *a* lattice parameter and 14.23 Å for *c* lattice parameter with a volume of 102.4 Å^3^. Similar lattice parameters were reported for polycrystalline NMC, calculated *via* Rietveld refinement from neutron diffraction data.^28^ Even though interfacial strain and slight differences in the stoichiometry of the film can influence lattice parameters and unit cell volume,^29^ our results are in good agreement with the reported values for bulk NMC ceramics, meaning that the proposed calculation of unit cell parameters from RSMs is an effective method for determining the crystal structure and unit cell volume of NMC epitaxial thin films.

**Fig. 3:**
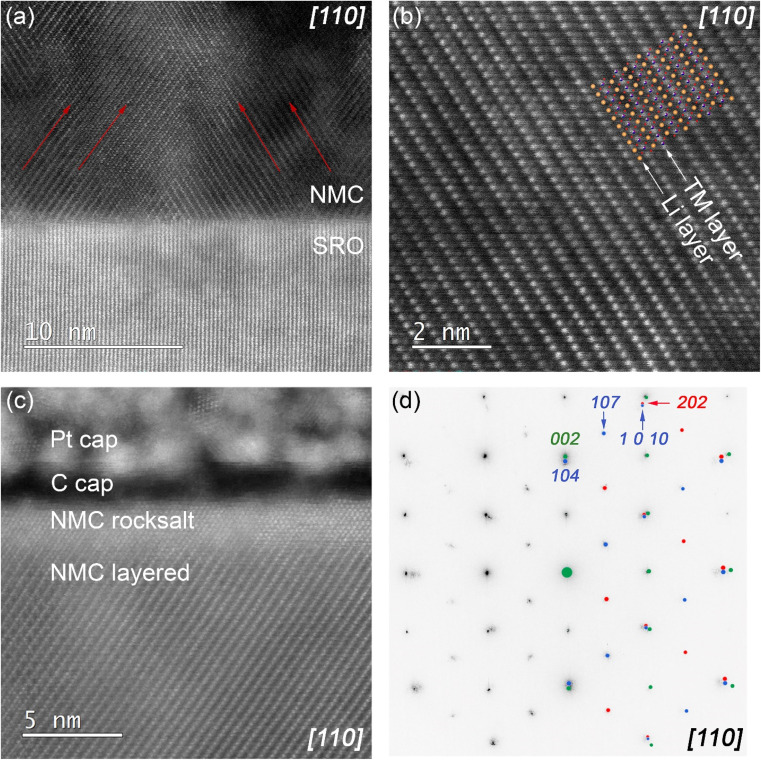
Scanning transmission electron microscopy analysis of NMC 111 thin film on SRO/Nb:STO (001), cut in [110] zone axis. (a) NMC 111/SRO interface revealed twinning in NMC 111 thin film; (b) alternating layers of lithium and transition metal ions in the NMC 111 layered structure; (c) presence of rock-salt layer on the surface of the NMC 111 thin film; (d) selected area electron diffraction of NMC 111/SRO/Nb:STO, including simulated patterns of NMC 111 twins (red and blue) and SRO/Nb:STO substrate (green).

To understand more about the growth mechanism and microstructure, thin film lamella was cut in [110] azimuth direction of the Nb:STO (001) substrate to reveal the layered structure of NMC 111. STEM image of the NMC 111 – SRO interface (Fig. 3a) revealed twinned domains in NMC 111 thin film (indicated with red arrows, parallel to the *a*-axis of the NMC unit cell), due to the tilted growth in out-of-plane direction and since 4-fold symmetry was proved with HRXRD, twinned domains in the crystal are also expected in [11̄0] direction. A high-resolution image of the NMC 111 layered structure (Fig. 3b), alternating between transition metal ions and lithium ions is consistent with the proposed structure determined with HRXRD (structural model of Nb:STO/SRO/NMC 111 thin film shown in Fig. S5†). However, STEM analysis of the thin film surface revealed the rock-salt structure of NMC 111 (Fig. 3c). The phase transition from layered to rock-salt structure might be initiated by exposure of the thin film to the ambient atmosphere or due to the sample preparation with FIB. The selected area diffraction patterns recorded in [110] azimuth direction (Fig. 3d) revealed diffraction spots of NMC 111, SRO and Nb:STO substrate. Simulated diffraction patterns for Nb:STO/SRO (green) and NMC 111 twins (red and blue) are consistent with the experimental patterns and clearly show twinning of the NMC 111 layers. Thus careful considerations must be taken before measuring asymmetric peaks with HRXRD to avoid overlapping of the crystal planes with the planes of the twin (*e.g.* (1 0 10) peak overlaps with the (202) peak from the twin), so (107) plane is chosen to measure lattice parameters accurately.

### 3.3. Surface analysis of LiNi_0.33_Mn_0.33_Co_0.33_O_2_ thin films

**Fig. 4:**
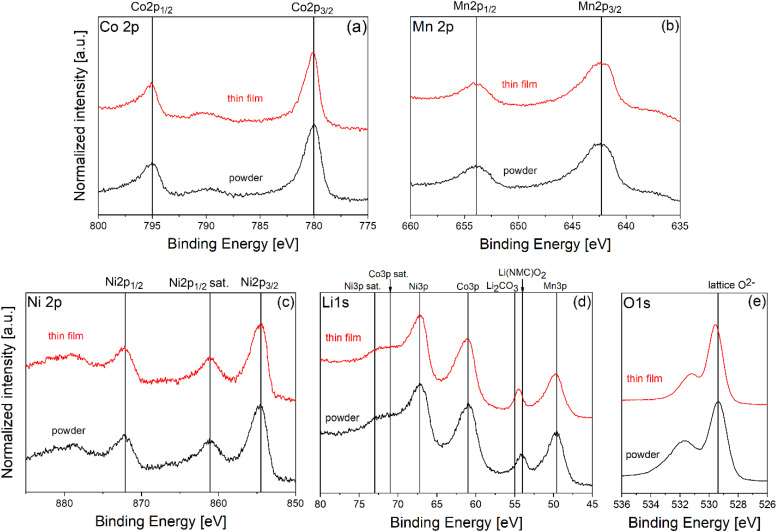
(a) Co 2p, (b) Mn 2p, (c) Ni 2p, (d) Li 1s and (e) O 1s photoelectron spectra of NMC 111 thin film, deposited at 0.01 mbar and annealed at 500 mbar oxygen pressure *vs.* stoichiometric NMC 111 powder as a reference.

The Li 1s, Ni 2p, Mn 2p, Co 2p and O 1s photoelectron spectra of NMC 111 calcined powder and NMC 111 thin film are shown in Fig. 4a–e. We use powder analysis as a reference for comparison with thin film photoelectron spectra. The lines in the graph are indicative binding energies of NMC 111 powder as reported elsewhere.^30^

Due to spin–orbit coupling, the Co 2p spectrum is split into two parts, attributed to Co 2p_3/2_ (780 eV) and Co 2p_1/2_ (795 eV). The position of main lines and a satellite peak (789.9 eV), associated with photoemission metal–ligand charge transfer, are indicative of Co^3+^ ions in NMC 111 thin film.^30^ Ni 2p region consists of Ni 2p_3/2_ and Ni 2p_1/2_ doublet at 854.5 eV and 872.1 eV, respectively, and two satellite peaks located at 861.1 eV and 878 eV. The peak positions of Ni 2p peaks are consistent with values reported for NMC 111 powders, where Ni ions are predominantly in Ni^2+^ oxidation state,^30,31^ but we cannot exclude the presence of Ni^3+^ ions, because for the Ni 2p_3/2_ spectrum of the thin film there is a higher signal-to-noise ratio, so there may be some broadening of the main line due to small amount of Ni^3+^. Moreover, Ni 2p_3/2_ main peak overlaps with Mn LM1 Auger peak.^32^ Mn 2p spectrum depicts two components located at 642.3 eV and 653.8 eV, consistent with Mn 2p_3/2_ and Mn 2p_1/2_ values for Mn^4+^ ions, respectively.^30^ XPS analysis of transition metals spectra confirmed the presence of Co^3+^, Ni^2+^ and Mn^4+^, consistent with previous attributions.

The binding energy range of 45–80 eV (Fig. 4d) includes Mn 3p, Li 1s, Ni 3p and Co 3p spectra. Mn 3p spectrum consists of a main peak at 49.7 eV, Ni 3p spectrum has a main peak at 67.3 eV and a satellite peak at 73.1 eV, while Co 3p spectrum shows a main peak at 61 eV and a satellite peak at 71 eV. Description of Li 1s spectrum requires two contributions; one for Li^+^ in the oxide lithium layers at 54 eV, while the other can be attributed to lithium residuals (Li_2_CO_3_ and LiOH) present on the surface at 55 eV.^30,32^ In addition to this, the presence of lithium residuals on the surface can be confirmed from O 1s spectra. O 1s region exhibits two peaks; the peak at 529.3 eV can be attributed to the O^2−^ framework in the NMC 111 and the one around 531.5 eV to the absorbed species and lithium residuals on the surface, as the sample was exposed to ambient atmosphere before XPS analysis.

**Fig. 5:**
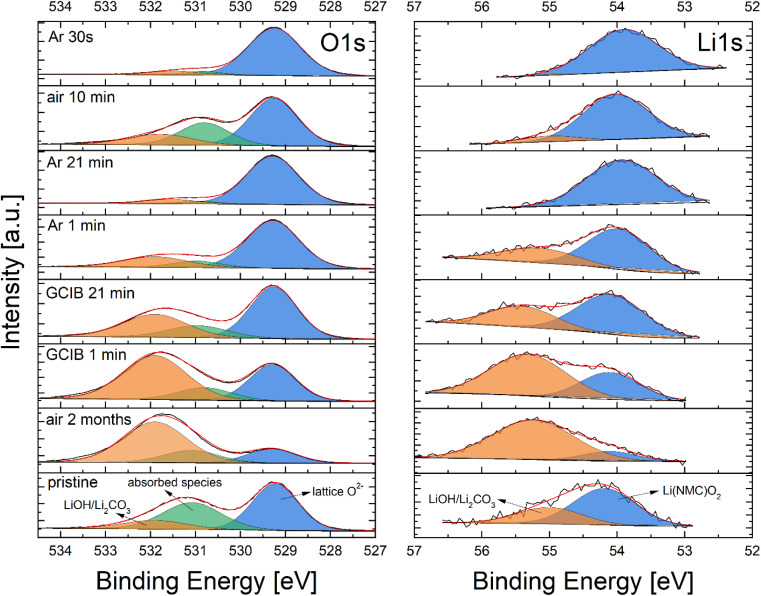
Evolution of O 1s and Li 1s photoelectron spectra of NMC 111 thin film after ambient atmosphere exposure and sputtering with argon ions.

Furthermore, NMC 111 thin film was aged for two months after XPS analysis to study the stability of the surface in the ambient atmosphere. The evolution of the NMC 111 surface after prolonged ambient exposure and a series of sputter cycles is shown on O 1s and Li 1s photoelectron spectra (Fig. 5). O 1s spectra were fitted with 3 peaks, attributed to lattice oxygen in NMC (529.3 eV), weakly absorbed species (531 eV) and lithium residuals (532 eV), while Li 1s spectra were fitted with two peaks, attributed to lithium in NMC layers (54 eV) and residual lithium on the surface (55 eV). After two months of ambient exposure, the contribution from lithium residuals on O 1s and Li 1s spectra starts to dominate in intensity, as moisture and CO_2_ in the ambient atmosphere enhance their formation. Afterward, the sample was cleaned with an argon cluster ion beam (GCIB) and argon ions to expose the underlying surface. A series of sputter cycles resulted in a clean surface of NMC 111, which was later exposed to ambient air for 10 minutes and measured again. Ambient atmosphere exposure resulted in the absorption of species that enhance the formation of lithium residuals, which suggests the high sensitivity of NMC 111 surfaces to moisture and CO_2_. Species absorbed in a short time of exposure were then quickly removed with argon ions.

### 3.4. Structural changes in delithiated and lithiated LiNi_1/3_Mn_1/3_Co_1/3_O_2_ thin films

**Fig. 6:**
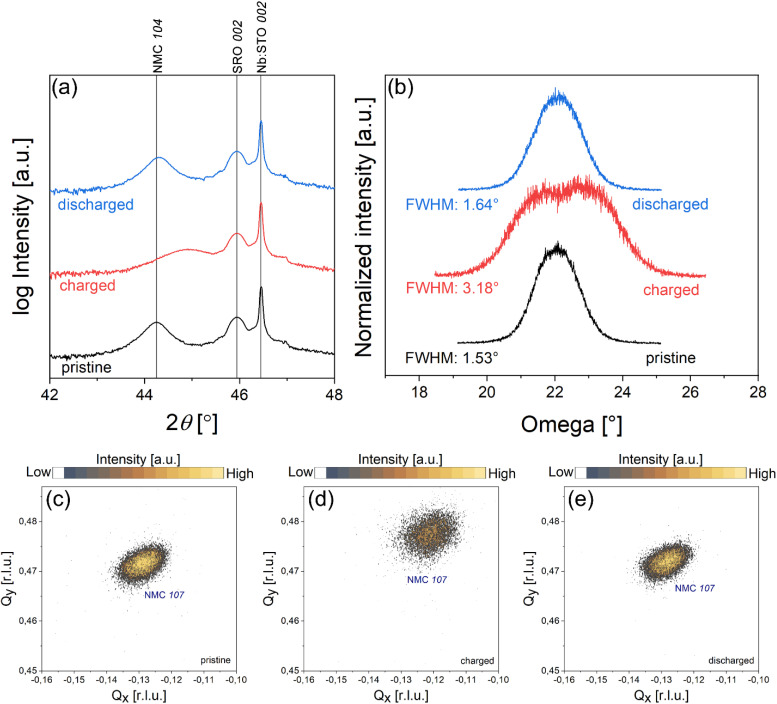
(a) Out-of-plane XRD pattern of pristine, charged and discharged NMC 111 thin film on SrRuO_3_/Nb:SrTiO_3_ (001) substrate. (b) Rocking curves on (104) peak of pristine, charged and discharged NMC 111 thin film. RSMs of (107) NMC 111 reflection in (c) pristine, (d) charged and (e) discharged state.

To understand how the structure of the NMC 111 changes after the delithiation and lithiation process, the thin film was charged to 4.2 V *vs.* Li/Li^+^ and discharged to 3.0 V *vs.* Li/Li^+^. The reversibility of lithium insertion in the thin film was analyzed by using the protocol described in the experimental section and presented in Fig. S6.† After the charge and discharge cycle, NMC 111 remains epitaxial and preserves the same orientation relationship to the substrate, but the shift and intensity of (104) reflection decrease in the charged state, which indicates a structural change in NMC 111 thin film (Fig. 6a). The volume change of NMC 111 at charged and discharged state was estimated from RSMs measured around (104) and (107) reflections of NMC 111 thin film (Fig. 6c–e), as discussed in the structural analysis chapter. Table 1 presents lattice parameters and respective volumes of pristine, charged, and discharged NMC 111 thin films. Results have confirmed that NMC 111 experiences a negative volume change with the delithiation process, related to shrinkage of *a* lattice parameter and elongation of *c* lattice parameter, as previously reported for polycrystalline NMC.^28^ Comparison of rocking curves on NMC 111 (104) peak (Fig. 6b) provides additional information about crystal structure at the charged state. We observed an increased broadening of the rocking curve in the delithiated NMC 111 compared to the pristine structure. The latter can be explained by the increase in mosaicity spread of NMC planes, which compensates for the negative volume change in the structure. Nevertheless, lithiation of the charged NMC 111 is reversible, as the positions of (104) and (107) reflections are comparable to a pristine thin film and the broadening of the NMC (104) peak reduces with lithiation. Based on these results, we can conclude that the delithiation induces negative volume change and broadening of crystal plane orientations in the epitaxial NMC 111 thin film.

**Table 1:** Lattice parameters and unit cell volumes of pristine, charged and discharged NMC 111 thin film

	*a* lattice parameter	*c* lattice parameter	Unit cell volume
Pristine	2.88 Å	14.23 Å	102.4 Å^3^
Charged	2.82 Å	14.24 Å	98.2 Å^3^
Discharged	2.88 Å	14.22 Å	102.1 Å^3^

### 3.5. Microstructural analysis of delithiated LiNi_1/3_Mn_1/3_Co_1/3_O_2_ thin films

To prove and correlate the structural changes that occur after delithiation, NMC 111 thin film was charged to 4.2 V *vs.* Li/Li^+^ identically as presented in Fig. S6,† followed by preparation of FIB lamella, cut in [110] azimuth direction of the Nb:STO (001) substrate, as described in the experimental section.

**Fig. 7:**
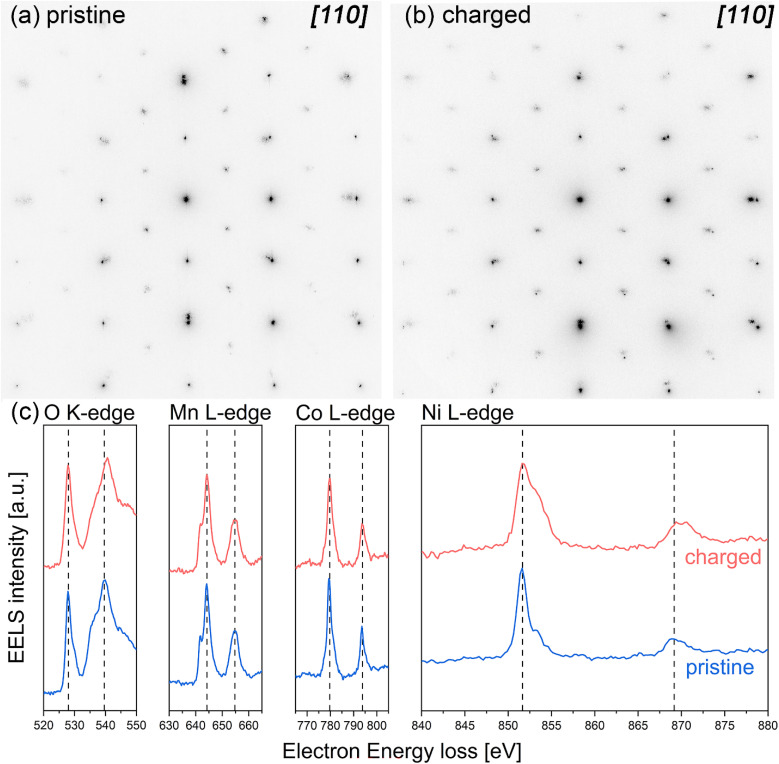
STEM analysis of charged NMC 111 thin film on SRO/Nb:STO (001), cut in [110] zone axis. Selected area electron diffraction of (a) pristine NMC 111 and (b) charged NMC 111, including SRO and the substrate; (c) electron energy loss O K-edge, Mn L-edge, Co L-edge and Ni L-edge spectra comparison of pristine and charged NMC 111 thin film.

Based on recorded diffraction patterns of pristine (Fig. 7a) and charged (Fig. 7b) NMC 111 thin film, corresponding with selected areas, presented in Fig. S7a and b,† no obvious change in the crystal symmetry is observed at the charged state and STEM analysis of NMC 111 thin film revealed twinned domains of the layered structure (Fig. S7c†), showing no deviation to pristine state. Nevertheless, the presence of the spinel phase in small amounts cannot be excluded.^21^ This result aligns closely with RSM data, which proved to be the more appropriate technique in terms of determining exact changes in distances between the planes. To understand the mechanism behind the delithiation process in NMC 111 thin films, EEL spectra were measured in the bulk of the pristine and charged thin film lamella, as shown in Fig. S7d and S7e,† respectively. Comparison of pristine and charged EEL spectra (Fig. 7c) showed the shift in O K-edge main peak position from 539.7 eV to 540.6 eV, which can be attributed to the change of local electronic structure of oxygen ions, thoroughly explained in the paper by Y. Koyama *et al.*^33^ The shift to higher energy is a consequence of nickel oxidation that increases the covalent bonding between nickel and oxygen, leading to the reduction of electron density at the oxygen ions. However, the shift in the peak position of the O K-edge pre-peak is not observed; this might be related to the beam sensitivity of the sample since the origin of the pre-peak can arise due to the radiation damage, observed in the complex oxides containing light alkali elements,^34^ impeding straight-forward explanation. Regarding the local electronic structure of transition metals at the charged state, there is no obvious shift in Mn L-edge and Co L-edge EEL spectra; in contrast, a broadening of Ni L-edge was observed. This change in Ni L-edge is related to the change in the nickel oxidation state; after lithium is extracted from the layered structure, nickel ions are oxidized to compensate for the charge and maintain charge neutrality in the delithiated structure, directly affecting the shape of Ni L-edge EEL spectrum, causing it to broaden towards higher energy values. Moreover, the change in intensity ratios between the *L*_3_ and *L*_2_ peaks of transition metals EEL spectra can indicate a change in the valence state.^35,36^ Determined *L*_3_/*L*_2_ intensity ratios in pristine and charged NMC thin film are presented in Table 2. Since the obvious change of *L*_3_/*L*_2_ intensity ratio occurs only on nickel EEL spectra, this further proves that the charge compensation mechanism in NMC is the oxidation of the nickel ions, which concurrently influences the local electronic structure of the oxygen ions.

**Table 2:** *L*_3_/*L*_2_ intensity ratios for pristine and charged NMC thin film, determined from EEL spectra by using a modified integral Mn, Ni and Co L3,2 white-line intensity ratios, calculated for each line sum spectrum

	Mn *L*_3_/*L*_2_ ratio	Co *L*_3_/*L*_2_ ratio	Ni *L*_3_/*L*_2_ ratio
Pristine	1.92	2.03	3.58
Charged	1.92	2.05	3.17

### 3.6. Electrochemical properties of LiNi_0.33_Mn_0.33_Co_0.33_O_2_ thin films

**Fig. 8:**
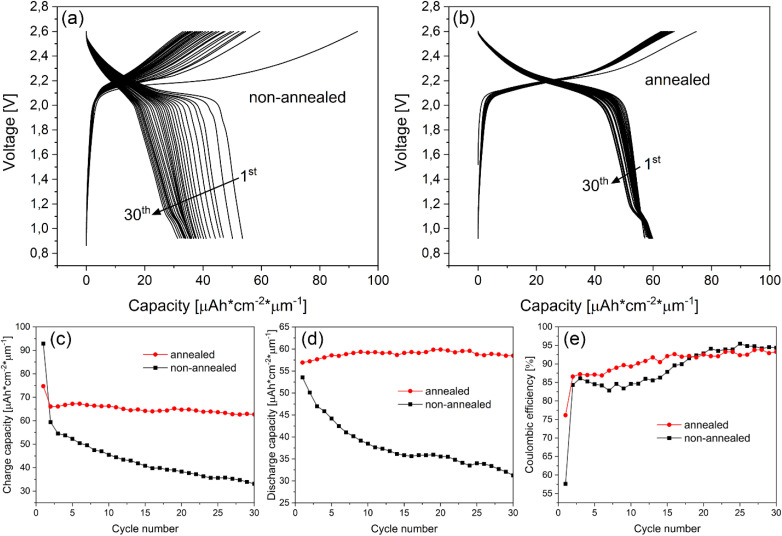
First 30 charge/discharge cycles of (a) non-annealed and (b) annealed NMC 111 thin films, deposited at 0.01 mbar and cycled *vs.* Li_4_Ti_5_O_12_ counter electrode with corresponding (c) charge capacity, (d) discharge capacity and (e) coulombic efficiency *vs.* cycle.

Charge and discharge curves of non-annealed and annealed NMC 111 thin films *vs.* Li_4_Ti_5_O_12_ are shown in Fig. 8a and b. The capacity of NMC 111 thin film is presented in specific volumetric units, calculated from thicknesses measured with XRR, assuming theoretical density. Based on the observed low first cycle coulombic efficiency (57.6%) of the non-annealed thin film (Fig. 8e), we consider a certain degree of oxygen non-stoichiometry, as there is not enough oxygen to form stoichiometric NMC 111 film at lower pressures. This can induce structural instability at the charged state,^6^ resulting in irreversible structural changes that hinder electrochemical performance. Annealing of NMC 111 thin films after deposition improved the coulombic efficiency of the first cycle (76.2%) (Fig. 8e) and long-term cycling stability, which can be attributed to additional oxygen incorporation in the NMC 111 thin film during cooling in high oxygen pressure, thus stabilizing the crystal structure. Moreover, the instability of the oxygen-deficient film is reflected as a constant decline in specific charge and discharge capacities (Fig. 8c and d). On the other hand, stable performance was observed for the annealed NMC 111 thin film after the first formation cycle that reached a specific discharge capacity around 60 μA cm^−2^ μm^−1^, which corresponds to 125 mA h g^−1^, meaning that the practical specific capacity is comparable to the polycrystalline NMC 111 cycled *vs.* LTO anode.^37^ The correlation of oxygen non-stoichiometry to the structural instability and electrochemical performance of NMC is discussed in the work of Bi *et al.*.^38^ They proved the dependence of oxygen content in the gaseous mixture during the annealing process on the degree of structural defects and electrochemical properties of NMC 811 powders. The sample prepared in pure oxygen exhibited the least amount of oxygen defects, leading to the highest discharge capacity, the best rate capability and the most stable cyclability. Even though this paper discusses NMC 811 composition, which is believed to be more susceptible to oxygen loss during high-temperature processing, the oxygen stoichiometry in NMC strongly depends on a processing technique. In another work, NMC 111 fibers are prepared *via* the electrospinning method, showing improved electrochemical properties of NMC 111 fibers, annealed in an oxygen atmosphere.^39^ In our case, oxygen pressure during PLD growth is moderate, which could produce oxygen-deficient films, so high-pressure annealing of NMC 111 thin films after deposition provides enough oxygen to be incorporated into the film, leading to improved electrochemical performance. Since there is a certain degree of capacity loss during the first cycle when comparing charge and discharge curves, we assume this is a result of kinetic limitations during discharge,^40^ irreversible structural changes at the charged state,^40^ or the formation of the solid electrolyte interphase layer on the surface of the cathode,^41^ which depends on the surface morphology and stoichiometry of the NMC 111 thin film.

**Fig. 9:**
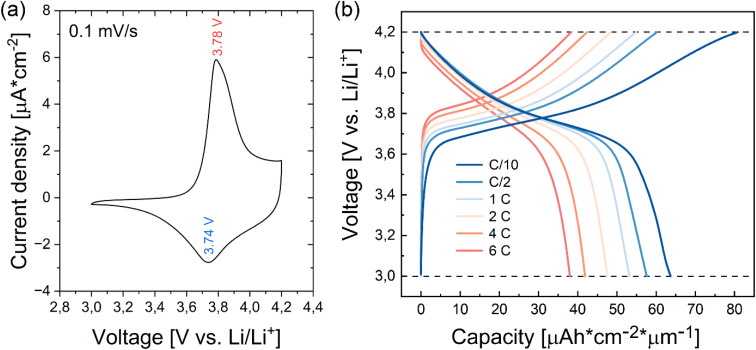
(a) Cyclic voltammetry of NMC 111 thin film cycled *vs.* Li/Li^+^. (b) Galvanostatic cycling curves of NMC 111 thin film *vs.* Li/Li^+^ at current densities of 0.4 μA cm^−2^, 2 μA cm^−2^, 4 μA cm^−2^, 8 μA cm^−2^, 16 μA cm^−2^ and 24 μA cm^−2^, corresponding to C/10, C/2, 1C, 2C, 4C and 6C, respectively.

To further elucidate the overall electrochemical behavior of annealed NMC 111 thin film, cyclic voltammetry^42^ and galvanostatic cycling measurements^43^ are performed in the voltage range of 3.0–4.2 V *vs.* Li/Li^+^. A cyclic voltammogram, shown in Fig. 9a, displays a well-defined cathodic peak at 3.78 V and an anodic peak at 3.74 V. Those two features are characteristic of NMC 111 that undergoes the process of lithium-ion insertion/deinsertion,^12^ coupled to the redox reaction of nickel ions, which aligns well with the observed nickel oxidation in NMC 111 thin film, charged up to 4.2 V. Moreover, the appearance of only one redox peak in the voltage range of 3.0–4.2 V suggests the absence of phase transitions from hexagonal to monoclinic structure.^44^ The effect of applied current density on the specific capacity was tested *via* galvanostatic cycling of NMC 111 thin film (Fig. 9b). At a current density of 0.4 μA cm^−2^, corresponding to the rate of C/10, NMC 111 thin film achieved a specific capacity of 63.7 μA cm^−2^ μm^−1^ (133.5 mA h g^−1^), similar to the annealed NMC 111 thin film, cycled *vs.* LTO anode. The capacity gradually declined with increasing rates, reaching 57.6 μA cm^−2^ μm^−1^ (120.8 mA h g^−1^) at C/2, 53.2 μA cm^−2^ μm^−1^ (111.5 mA h g^−1^) at 1C, 47.5 μA cm^−2^ μm^−1^ (99.6 mA h g^−1^) at 2C, 41.9 μA cm^−2^ μm^−1^ (87.8 mA h g^−1^) at 4C and 38.0 μA cm^−2^ μm^−1^ (79.7 mA h g^−1^) at 6C. Gradual fading of the specific capacity at faster charge/discharge rates is caused by kinetic restrictions of active material since diffusivity coefficient of lithium ions is at least one order of magnitude lower than electronic conductivity in stoichiometric NMC 111, while even larger differences are observed when lithium is removed from the structure.^45^ Even though the diffusion length for lithium ion transport is short, the rate-controlling step for fast charge and discharge is the solid-state diffusion of lithium ions.

## 4. Conclusions

Epitaxial NMC 111 with (104) out-of-plane orientation deposited on SRO/Nb:STO (001) substrates by pulsed laser deposition proved to be fully relaxed with 4-fold symmetry and 45° tilt of the unit cell with respect to the substrate. Lattice parameters and the corresponding volume of NMC 111 unit cell are calculated from (104) and (107) reflections of the thin film, measured with HRXRD. Microstructure analysis revealed twinned domains in the layered structure of NMC 111 with the presence of a rock-salt phase on the surface. Surfaces of NMC 111 thin films are prone to absorb moisture and form lithium residuals on the surface when in contact with the ambient atmosphere, enhancing the formation of non-desirable phases. After delithiation of NMC 111 thin films, nickel ions are oxidized to maintain charge neutrality, while the crystal structure experienced negative volume change and increased mosaicity, which proved to be a reversible process. Poor electrochemical performance of oxygen-deficient NMC 111 thin films is a consequence of the structural instability in the charged state which can be improved by cooling the films in high oxygen pressure after deposition. Cyclic voltammetry displays well-defined cathodic and anodic peaks, while a decrease of specific capacity from 63.7 μA cm^−2^ μm^−1^ at C/10 to 38.0 μA cm^−2^ μm^−1^ at 6C revealed the kinetic limitations of NMC 111 at higher cycling rates.

## Data availability

The authors confirm that the data supporting the findings of this study are available within the article and ESI file.† The data supporting the findings of this study were collected during the completion of a doctoral dissertation and are currently stored on a local computer. Due to data protection, they are not available in a public repository. However, the data may be accessed upon reasonable request. Contact Blaž Jaklič blaz.jaklic@ijs.si.

## Author contributions

The manuscript was written through the contributions of all authors. All authors have given approval to the final version of the manuscript. Blaž Jaklič: writing – original draft, conceptualization, formal analysis, investigation, methodology, visualization. Jan Žuntar: investigation, formal analysis. Elena Tchernychova: investigation, formal analysis, validation, project administration. Gregor Kapun: investigation, formal analysis. Martin Šala: investigation, formal analysis. Robert Dominko: writing – review and editing, validation, resources, supervision, project administration, funding acquisition. Matjaž Spreitzer: writing – review and editing, validation, conceptualization, resources, supervision, project administration, funding acquisition.

## Conflicts of interest

There are no conflicts to declare.

## Supplementary Material

RA-015-D4RA08924C-s001
