# Supplementary material for: Structural changes and mechanisms of reversible electrochemical lithium-ion cycling in (104) oriented LiNi1/3Mn1/3Co1/3O2 thin film cathodes prepared by pulsed laser deposition
Source: RSC Adv. 2025 Apr 7;15(14):10787–800. doi: 10.1039/d4ra08924c (PMC11973892; doi:10.1039/d4ra08924c)
Supplement: RA-015-D4RA08924C-s001 [file RA-015-D4RA08924C-s001.pdf]

Supplementary Information (SI) for RSC Advances.
This journal is © The Royal Society of Chemistry 2025

Supporting information

Structural changes and mechanisms of reversible electrochemical lithium-ion cycling in (104) oriented $LiNi_{1/3}Mn_{1/3}Co_{1/3}O_2$ thin film cathodes prepared by pulsed laser deposition

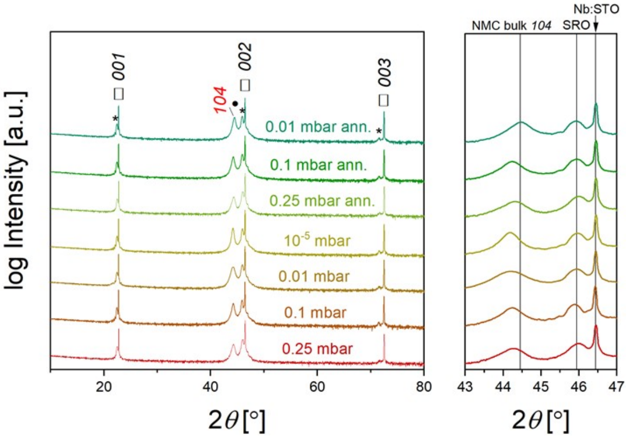

Figure S1. Out-of-plane XRD patterns of NMC 111 thin films on $SrRuO_3$/Nb:$SrTiO_3$ *(001)* substrate, deposited/cooled down at different oxygen pressures. * $SrRuO_3$ peaks, □ Nb:$SrTiO_3$ peaks, • NMC 111 peak.

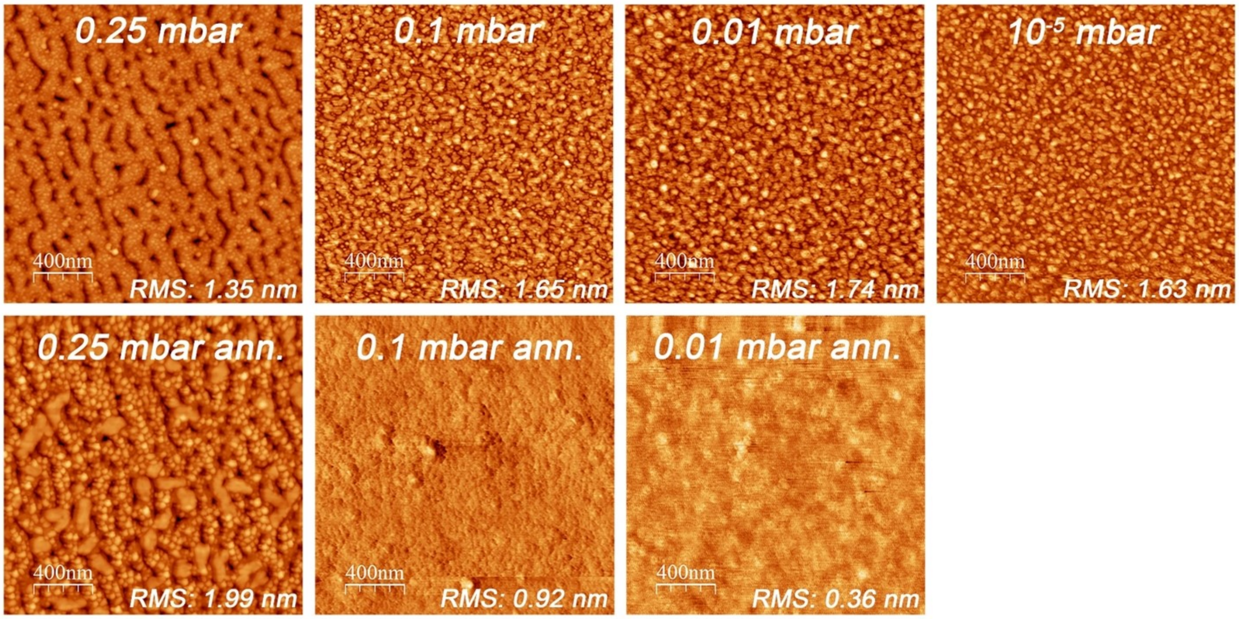

Figure S2. AFM images of NMC 111 thin films on $SrRuO_3$/Nb:$SrTiO_3$ *(001)* substrate, deposited/cooled down at different oxygen pressures.

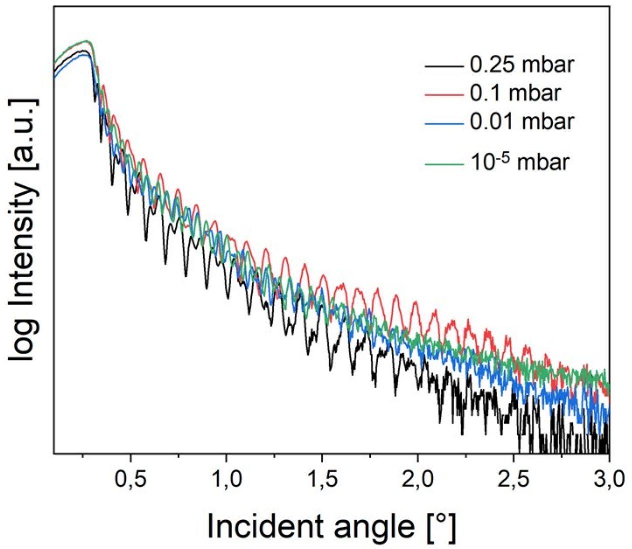

Figure S3. X-ray reflectometry patterns of NMC 111 thin films on $SrRuO_3$/Nb:$SrTiO_3$ *(001)* substrate, deposited at oxygen pressures ranging from 0.25 mbar to 10^{-5} mbar.

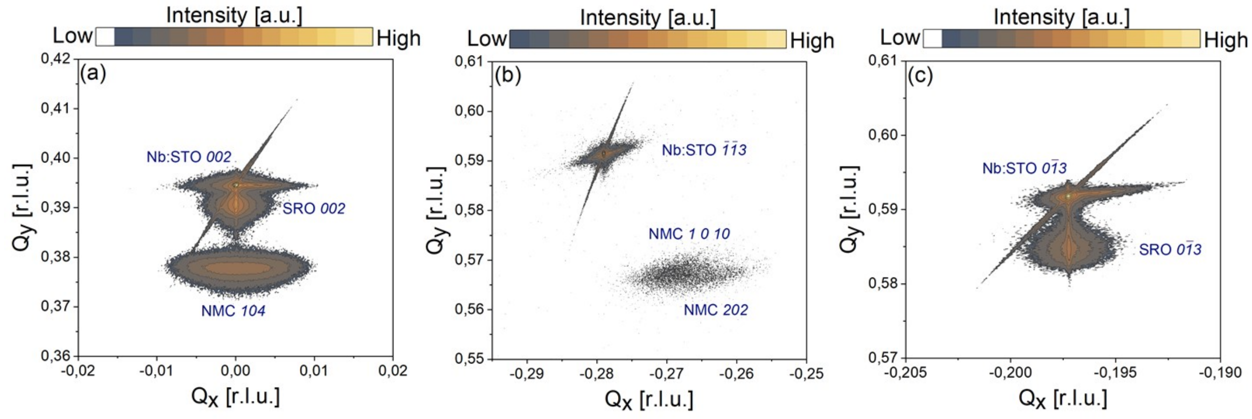

Figure S4. Reciprocal space maps around (a) symmetrical *(002)* reflection, (b) asymmetrical $(\bar{1}\bar{1}3)$ reflection and (c) asymmetrical $(0\bar{1}3)$ reflection of Nb:STO substrate.

To correctly determine lattice parameters and volume of NMC 111 thin films, Q_x and Q_y values were first transformed to normalized reciprocal lattice vectors (NRLV) using **Equation S1**, where λ = 1.5406 Å (wavelength of Cu-Kα) :

$$Q_{x,y(normalized)} = \frac{2Q_{x,y}}{\lambda}\#(S1)$$

D-spacings of *(104)* and *(107)* planes were calculated from NRLV using **Equation S2**:

$$\frac{1}{d_{(104)/(107)}} = \sqrt{Q_{x(104)/(107)}^{2} + Q_{y(104)/(107)}^{2}}\#(S2)$$

To calculate the *a* and *c* lattice parameters from the d-spacing of *(104)* and *(107)* planes, **Equation S3** which correlates d-spacing and lattice parameters in hexagonal unit cell was used:

$$\frac{1}{d^2} = \frac{4}{3}\left(\frac{h^2 + hk + k^2}{a^2}\right) + \frac{l^2}{c^2}\#(S3)$$

Based on this correlations, *a* and *c* lattice parameters were calculated from system of two equations **Equation S4** and **Equation S5**:

$$\frac{1}{d_{(104)}^{2}} = \frac{4}{3a^2} + \frac{16}{c^2}\#(S4)$$

$$\frac{1}{d_{(107)}^{2}} = \frac{4}{3a^2} + \frac{49}{c^2}\#(S5)$$

Volume of the NMC 111 hexagonal unit cell was calculated from **Equation S6**:

$$V_{NMC\,111} = \frac{\sqrt{3}}{2}a^2c\#(S6)$$

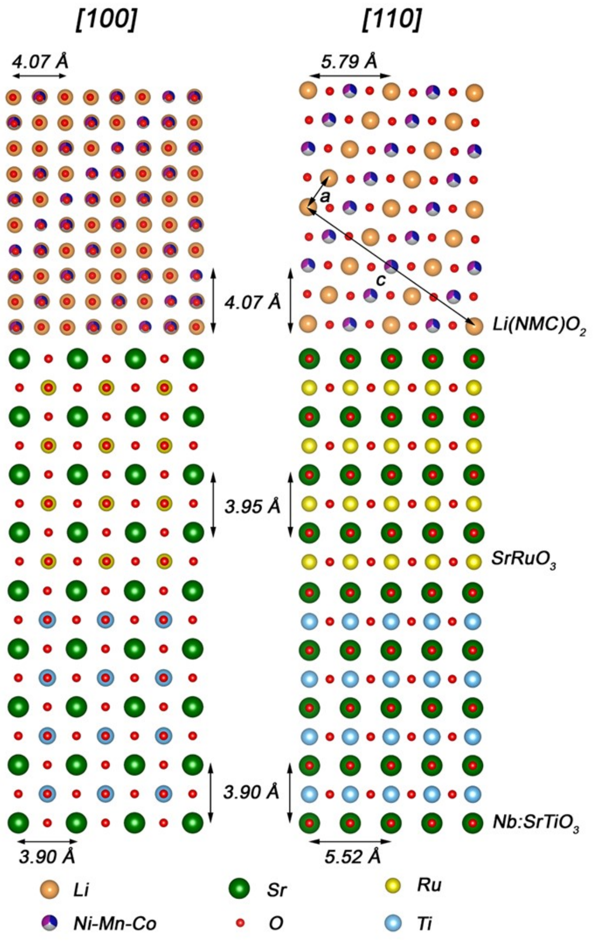

Figure S5. Structural model of NMC 111/SRO/Nb:STO thin film, viewed in *[100]* and *[110]* zone axis of the substrate.

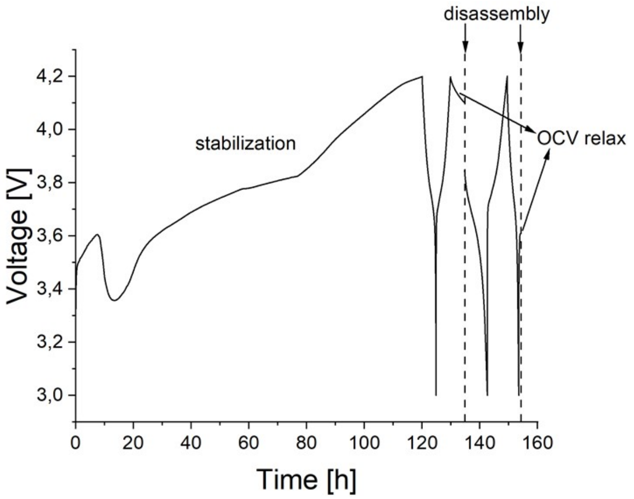

Figure S6. Electrochemical charging and discharging process of NMC 111 thin film vs. Li^+ anode.

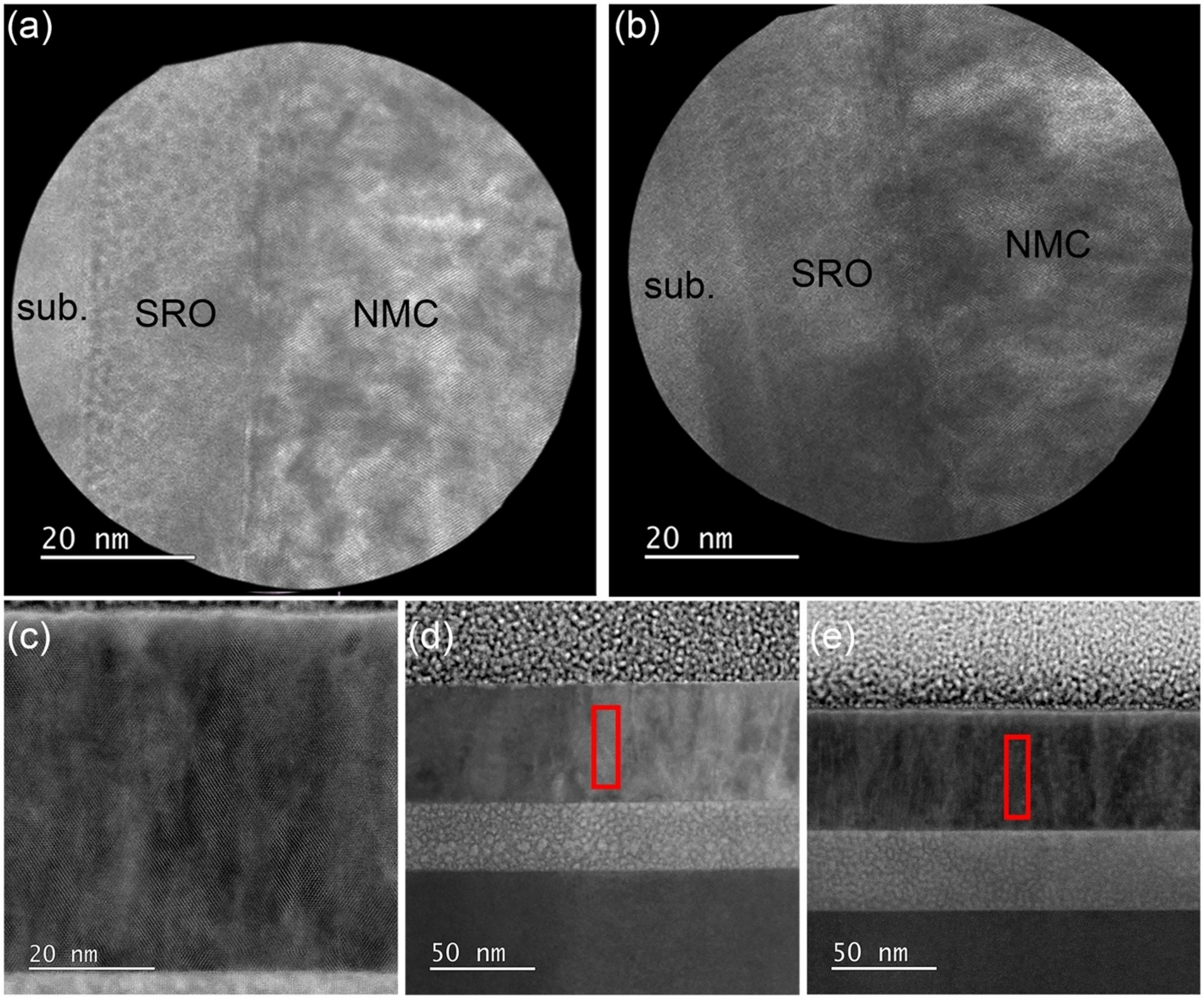

Figure S7. Selected areas for electron diffraction on (a) pristine and (b) charged NMC 111/SRO/Nb:STO heterostructure; (c) STEM micrograph of charged NMC 111 thin film; Selected areas for EELS analysis on (d) pristine and (e) charged NMC thin film.

Preparation of $Li_4Ti_5O_{12}$ counter electrodes

To avoid short circuits due to the lithium dendrite formation and thus provide a stable cycling environment for the NMC thin film working electrodes, bulk $Li_4Ti_5O_{12}$ (LTO) electrodes were

prepared in-house to be used as counter electrodes. The electrodes were prepared using a formulation of 80 wt. % $Li_4Ti_5O_{12}$ powder, 10 wt. % Super C65 carbon black (Imerys) and 10 wt. % poly(vinylidene difluoride) (PVdF, Sigma Aldrich) for the solid content, to which *N*-methyl pyrrolidone (NMP, Supelco) was added as a solvent (3.4 mL/g of solids). The contents were then homogenized in a planetary ball mill (30 min, 300 rpm), cast on 20 µm thick Cu foil with a doctor blade (300 µm wet thickness) and dried overnight at reduced pressure (5 mbar) in a vacuum oven at 90 °C. Afterward, circular 16 mm diameter samples were cut out, pressed in a hydraulic press (30 seconds at 1 tonne), dried overnight again and transferred to an argon-filled glove box. To avoid any overlap of the bulk electrode with the sharp edges of the thin film electrodes, the transferred samples were then punched to a smaller diameter than the working electrodes (6 mm LTO diameter for 10x10 mm NMC electrode).

Pouch-type cells were assembled in the glove box with the inner stack consisting of the as-prepared LTO electrode, a glass microfiber filter paper GF/A separator (Whatman, 260 µm, 18 mm diameter) and Li metal foil (110 µm, FMC) as the counter electrode. After the addition of 90 µL of electrolyte (1 M $LiPF_6$ in EC/ DEC = 1:1 vol. (LP40, Sigma-Aldrich)), the stack was enclosed in Triplex foil with Cu foil strip contacts and sealed under vacuum.

The pouch cells were galvanostatically precycled (C/10 current, 1,0 – 2,5 V) for two discharge-charge cycles, then discharged to approximately 60 % state of charge (SoC) (**Figure S8**) and disassembled in an Ar-filled glove box. As the voltage plateau for the LTO at this SoC is very flat and the discharge capacity of the LTO electrodes was roughly two orders of magnitude larger than that of the NMC thin-film electrodes, this allowed us to use the precycled LTO electrodes as a constant-potential counter electrode for further electrochemical testing of NMC thin-film electrodes.

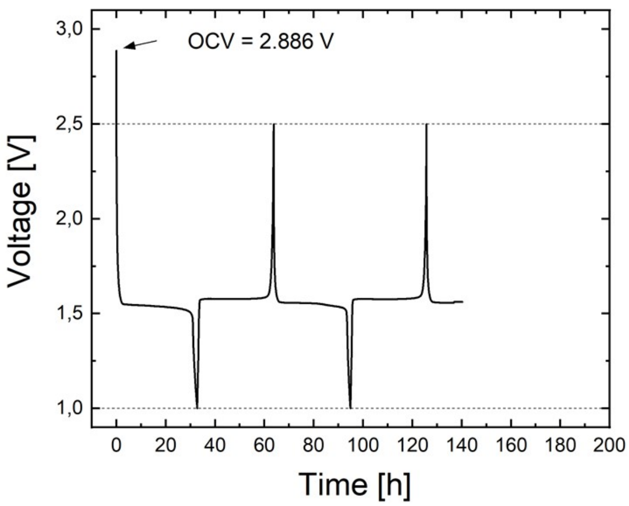

Figure S8. Electrochemical precycling process of $Li_4Ti_5O_{12}$ electrodes vs. Li^+ anode.
